# Printability in Multi-material Projection-Based 3-Dimensional Bioprinting

**DOI:** 10.34133/research.0613

**Published:** 2025-03-04

**Authors:** Chao-fan He, Tian-hong Qiao, Xu-chao Ren, Mingjun Xie, Qing Gao, Chao-qi Xie, Peng Wang, Yuan Sun, Huayong Yang, Yong He

**Affiliations:** ^1^ State Key Laboratory of Fluid Power and Mechatronic Systems & Liangzhu Laboratory, School of Mechanical Engineering, Zhejiang University, Hangzhou 310027, China.; ^2^ Key Laboratory of 3D Printing Process and Equipment of Zhejiang Province, College of Mechanical Engineering, Zhejiang University, Hangzhou 310027, China.; ^3^ School of Computer Science, Xi’an Shiyou University, Xi’an 710065, China.; ^4^ EFL-Tech, Suzhou Yongqinquan Intelligent Equipment Co., Ltd, Suzhou 215101, China.; ^5^ State Key Laboratory of Fluid Power and Mechatronic Systems, School of Mechanical Engineering, Zhejiang University, Hangzhou 310027, China.; ^6^ The Second Affiliated Hospital of Zhejiang University, Zhejiang University, Hangzhou 310027, China.

## Abstract

Accurately reconstructing the intricate structure of natural organisms is the long-standing goal of 3-dimensional (3D) bioprinting. Projection-based 3D printing boasts the highest resolution-to-manufacturing time ratio among all 3D-printing technologies, rendering it a highly promising technique in this field. However, achieving standardized, high-fidelity, and high-resolution printing of composite structures using bioinks with diverse mechanical properties remains a marked challenge. The root of this challenge lies in the long-standing neglect of multi-material printability research. Multi-material printing is far from a simple physical assembly of different materials; rather, effective control of material interfaces is a crucial factor that governs print quality. The current research gap in this area substantively hinders the widespread application and rapid development of multi-material projection-based 3D bioprinting. To bridge this critical gap, we developed a multi-material projection-based 3D bioprinter capable of simultaneous printing with 6 materials. Building upon this, we established a fundamental framework for multi-material printability research, encompassing its core logic and essential process specifications. Furthermore, we clarified several critical issues, including the cross-linking behavior of multicomponent bioinks, mechanical mismatch and interface strength in soft–hard composite structures, the penetration behavior of viscous bioinks within hydrogel polymer networks, liquid entrapment and adsorption phenomena in porous heterogeneous structures, and error source analysis along with resolution evaluation in multi-material printing. This study offers a solid theoretical foundation and guidance for the quantitative assessment of multi-material projection-based 3D bioprinting, holding promise to advance the field toward higher precision and the reconstruction of more intricate biological structures.

## Introduction

Three-dimensional (3D) bioprinting, as a specialized form of 3D-printing technology, utilizes biomaterial inks or bioinks to construct 3D bioactive structures [1]. Given the intrinsic differences among individual organisms, the complexity of natural tissues/organs, and the demand for rapid prototyping, bioprinting has demonstrated marked advantages in the biomedical field, revolutionizing the production mode of tissue engineering [2–4]. Currently, biomaterials can be broadly categorized as synthetic and natural [5]. While synthetic materials offer tunable properties, natural materials, particularly those derived from the extracellular matrix, have gained increasing attention due to their ability to closely mimic the cellular microenvironment [6,7]. However, single-material bioprinting faces limitations in the fabrication of functional organs, primarily due to the inherent complexity of natural organs, which often consist of combinations of tissue structures that are physically distinct but biochemically interconnected. Examples include the multilayered structure of blood vessels [8] and the millions of hepatic lobules in the liver [9]. These heterogeneous structures not only coordinate biochemical interactions among different cell populations but also provide structural foundations for various tissue functions [10]. Therefore, utilizing multi-materials to construct spatially heterogeneous biological structures is a crucial step in advancing the development of bioprinting [11].

Currently, while multinozzle extrusion-based printing (EBP) offers a relatively accessible and effective approach to multi-material printing, the inherent limitations of nozzles typically confine the resolution of EBP to around 100 μm. Although embedded EBP can achieve higher resolutions, it does so at the expense of design flexibility. Moreover, the fundamental printing unit in EBP is a 1-dimensional filament. Most studies have characterized resolution by measuring filament width (positive resolution) [12]. However, EBP often exhibits more pronounced limitations in terms of negative resolution (assessing pore structures) [13], lateral resolution (characterizing in-plane printing errors) [14], and vertical resolution (evaluating interlayer errors) [15].

Projection-based 3D-printing (PBP) technology employs a spatial light modulator, such as a digital micromirror device (DMD) or liquid crystal display, to modulate a light source and generate a dynamic optical pattern [16], enabling simultaneous solidification across the entire 2-dimensional (2D) plane. Although the basic printing unit in PBP is a 2D plane, the minimum voxel size is defined by individual pixels within the optical pattern. Taking a commonly used DMD as an example, it offers a resolution of 1,920 × 1,080 [17] with individual mirror sizes ranging from 5 to 10 μm, enabling PBP to achieve synchronous construction of 2 million voxels in a single exposure, with an optical resolution of 10 μm (using a ×1 objective lens). PBP exhibits a superior resolution-to-manufacturing time (RTM) ratio (≈2) [18], distinguishing it from other 3D-printing technologies. This unique combination of precision and efficiency endows projection-based 3D bioprinting (PBBP) with great potential for advancement, particularly in comparison to the most accurate 2-photon polymerization (RTM ≈ 0.05) and the most commonly employed EBP (RTM ≈ 0.5).

Nonetheless, PBP relies on photopolymerization to solidify liquid materials, complicating and challenging the handling of material interfaces. Multi-material PBP technology faces numerous challenges in material interface control, including ink cross-contamination, inadequate bonding strength, compatibility of material combinations, and variations in photoresponsive characteristics. Many fundamental questions in this field remain unanswered.

While several studies have proposed implementation strategies for multi-material PBP [19–24], there is currently a complete absence of systematic research on printability. The long-standing neglect of printability research, coupled with the stringent requirements for biocompatibility, has severely limited the practical applications of multi-material PBBP. This technology is currently confined to the preliminary stages of proof of concept, and there are many limitations in terms of model design freedom, manufacturing flexibility, and applicability to various scenarios. To bridge this gap and promote widespread adoption of multi-material bioprinting, it is crucial to establish a standardized and universally applicable evaluation system and process standards.

**Fig. 1.**
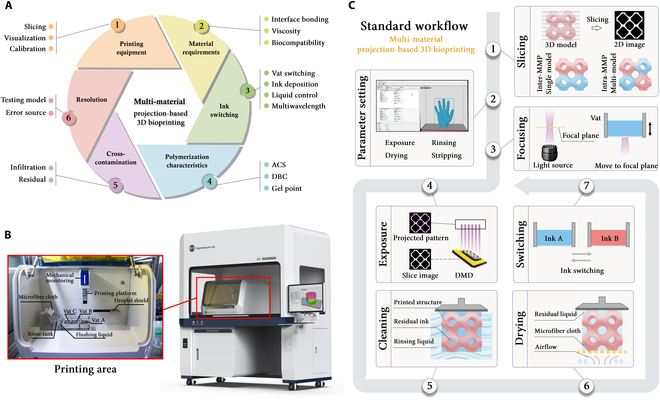
(A) Key elements of multi-material projection-based 3-dimensional bioprinting (PBBP). (B) Multi-material PBBP printer design. (C) Standard workflow of multi-material PBBP. ACS, average cross-linking step; DBC, double bond conversion; 3D, 3-dimensional; 2D, 2-dimensional; Inter-MMP, interlayer multi-material 3D printing; Intra-MMP, intralayer multi-material 3D printing; DMD, digital micromirror device.

Considering this, we summarized the core process requirements related to multi-material PBBP, which can be categorized into ink switching, polymerization characteristics, material requirements, cross-contamination, print resolution, and printing equipment (Fig. 1A). Subsequently, we developed a versatile multi-material 3D printer and conducted a systematic study on the printability of multi-material PBBP based on this device. Initially, we investigated the photo-cross-linking behavior of multicomponent hydrogels. Furthermore, we explored the variations in interfacial bonding strength and designed modifications to the interfacial microstructure and transition zone, providing feasible enhancement solutions. Notably, not all material combinations are suitable for multi-material printing. Therefore, we established multi-material printability standards to assess the feasibility of specific material combinations. Additionally, to guide the optimization of printing parameters, we proposed a multi-material print resolution evaluation model. This work is expected to pave the way for achieving high-resolution and efficient multi-material bioprinting.

## Results

### System design of a multi-material PBBP printer

The development of a highly automated, versatile multi-material 3D bioprinter is essential for achieving standardization, high fidelity, and efficiency. Current projection-based multi-material printers are predominantly modified from single-material printers, resulting in inadequacies in optimizing for multi-material applications. This limits the versatility of model design, manufacturing capabilities, and adaptability to various application scenarios. To address these issues, we innovatively developed a user-friendly, versatile multi-material 3D bioprinter with a 25-μm optical resolution and support for up to 6-material printing (Fig. 1B, Figs. S1 and S2, and Movie S1). This printer is equipped with a dedicated operating system, a mechanical monitoring system, a visual observation module, a laser calibration device, a fluid rinsing system, a negative-pressure drying device, and a flexible peel-off mechanism. The printing area is installed in a standard clean bench to ensure a sterile environment throughout the printing process. The projection system employs a 1,920 × 1,080 resolution DMD chip, enabling a maximum print size of 48 × 27 × 50 mm with a minimum layer thickness of 5 μm. Given the complexity of multi-material printing processes, which often require manual fine-tuning, we specifically introduced a visual system to allow for real-time monitoring and immediate adjustments during printing (Movie S2).

#### Complexity analysis of multi-material PBP

Unlike inkjet or extrusion printing that assembles from 0-dimensional points or 1-dimensional lines, PBP distinguishes itself through the direct projection and solidification of 2D patterns, thereby enhancing printing efficiency by reducing assembly time and improving layer uniformity. However, this characteristic also categorizes all multi-material PBP into 2 main types: interlayer multi-material 3D printing (Inter-MMP) and intralayer multi-material 3D printing (Intra-MMP) (Fig. S3). Inter-MMP is simpler and minimizes complexity, whereas intra-MMP involves more frequent switching and control challenges, increasing complexity and demanding higher accuracy.

Inter-MMP employs a singular printing material within each layer while sequentially switching to different materials for subsequent layers. This approach maintains the fundamental printing unit but utilizes printing units with different materials during the layer-by-layer assembly process. Consequently, Inter-MMP exhibits relative ease of implementation and minimal impact on overall printing complexity. The complexity of Inter-MMP can be expressed by the following equation:

$$T_{\text{inter}}=\sum_{l=1}^{x}\left({\mathrm{ET}}_{l}+\;{\mathrm{LT}}_{l}+\;{\mathrm{IT}}_{l}\right)+\sum_{s=1}^{y}\left({\mathrm{MT}}_{s}+\;{\mathrm{CT}}_{s}\right)$$ (1)

where x is the number of layers; ${\mathrm{ET}}_{l}$, ${\mathrm{LT}}_{l}$, and ${\mathrm{IT}}_{l}$ are the exposure time, lift time, and interval time per layer, respectively; y is the number of switches; and ${\mathrm{MT}}_{s}$ and ${\mathrm{CT}}_{s}$ are the platform move time and cleaning time, respectively.

Intra-MMP utilizes multiple materials within a single layer, with each material occupying a distinct subregion. It involves more switching times and control difficulties, making the overall printing process more complex and requiring higher printing and positioning accuracy. It is worth noting that the Intra-MMP method is downward compatible with the Inter-MMP method. The complexity of Intra-MMP can be expressed by the following equation:

$$T_{\text{intra}}=\sum_{l=1}^{x}\sum_{m=1}^{y}\left({\mathrm{ET}}_{\left(l,m\right)}+{\mathrm{LT}}_{\left(l,m\right)}+{\mathrm{IT}}_{\left(l,m\right)}+{\mathrm{MT}}_{\left(l,m\right)}+{\mathrm{CT}}_{\left(l,m\right)}\right)$$ (2)

where x is the number of layers; y is the number of materials used; and ${\mathrm{ET}}_{\left(l,m\right)}$, ${\mathrm{LT}}_{\left(l,m\right)}$, and ${\mathrm{IT}}_{\left(l,m\right)}$ are the exposure time, lift time, and interval time per material per layer, respectively. If a material is not used in a layer, the corresponding time is 0; ${\mathrm{MT}}_{\left(l,m\right)}$ and ${\mathrm{CT}}_{\left(l,m\right)}$ are the platform move time and cleaning time per ink switching per layer, respectively. If a material is not switched after printing within that layer, the corresponding time is 0.

The printing complexity of Inter-MMP is primarily influenced by the number of layers and the frequency of ink switching. Notably, even with ink switching occurring every 2 layers, the overall printing complexity does not increase obviously. Consequently, the complexity of Inter-MMP is essentially comparable to that of single-material PBP. Due to early hardware limitations, some research groups adopted a manual switching approach [25] to achieve Inter-MMP in their initial work. Although this method is suitable for constructing layered structures, such as skin repair [25], corneal regeneration [26], and microfluidic chip fabrication [27], it enables multi-material printing in only 1 dimension, limiting the ability to accurately mimic the complexity of natural tissues. In contrast, Intra-MMP can construct more complex geometric shapes and structures, such as those with intertwined, nested, or overhanging features, which align more closely with the complex characteristics of real biological tissues. However, this also results in a marked increase in printing complexity. In extreme cases, Intra-MMP may take tens of times longer than single-material printing. Therefore, research on its printability becomes crucial. This advancement will pave the way for the widespread use of multi-material printing and its transformative potential across various applications.

#### Approaches of ink switching

Ink switching is vital in multi-material printing, often using a bottom-up approach [26–29] to avoid constraints during switching. This method places the light source below the lifting printing platform. Established solutions include vat switching [25,30,31], ink deposition [32,33], fluidic control [34,35], and multiwavelength photopolymerization [19] (Fig. S4). Vat switching is the most versatile approach but faces the challenge of cross-contamination due to incomplete cleaning. Ink deposition, a simplified version of vat switching, is prone to contamination and is not suitable for bioprinting scenarios. Fluidic control methods, which switch inks by setting inlets and outlets at both ends of the vat, have high viscosity requirements for the ink and result in huge waste. Multiwavelength photopolymerization technology mixes inks together and selectively cures specific components, avoiding the need for a cleaning step; however, the selection range of inks and initiators is highly limited, and cross-contamination cannot be avoided. In this study, a cleaning solution based on vat switching combined with fluid control rinsing and negative-pressure-assisted capillary adsorption is proposed to minimize cross-contamination. This study systematically explores the complex “print–switch–clean–dry–reprint” process in multi-material printing, laying the foundation for establishing a standard framework for multi-material printing.

#### Standard workflow of multi-material PBBP

Multi-material bioprinting consists of 2 stages: the preparation stage and printing stage. Preparation involves 3D model slicing, setting of print parameters, and focusing, done once per task. Printing, which includes exposure, washing, drying, and ink switching, is repeated multiple times. The process starts with acquiring 3D data and slicing them into 2D images using specific software, with different strategies for Inter-MMP and Intra-MMP (Fig. S5). Optimizing printing parameters, such as exposure, cleaning, and detachment settings, is crucial for print quality. After setting parameters, the printing platform moves to the focal plane, where a digital pattern is projected to photopolymerize the bioink. The platform then either rises to print the next layer if the ink is the same or cleans and dries the structure if it changes. Depending on the multi-material printing type, the platform either rises for the next layer after vat switching (Inter-MMP) or solidifies the next subplane within the layer (Intra-MMP). This repeats until all structures are printed (Fig. 1C and Fig. S6).

### Polymerization characteristics of multi-material bioinks

Hydrogels, with their high water content, biocompatibility, and degradability, are widely used in bioinks. Photocurable hydrogels are usually synthesized by the chemical reaction of biomaterials with methacrylic anhydride or acryloyl chloride, such as gelatin methacryloyl (GelMA) [4] and hyaluronic acid methacryloyl (HAMA) [36], and poly(ethylene glycol) diacrylate (PEGDA) [37]. Currently, there is still a lack of deep understanding of the photopolymerization behavior of multi-material bioinks, especially the photopolymerization kinetics and mechanisms at the interfaces. Therefore, the photopolymerization behavior and characteristics of multi-material bioinks were first investigated, taking HAMA and GelMA hydrogels, commonly used in bioprinting, as examples.

**Fig. 2.**
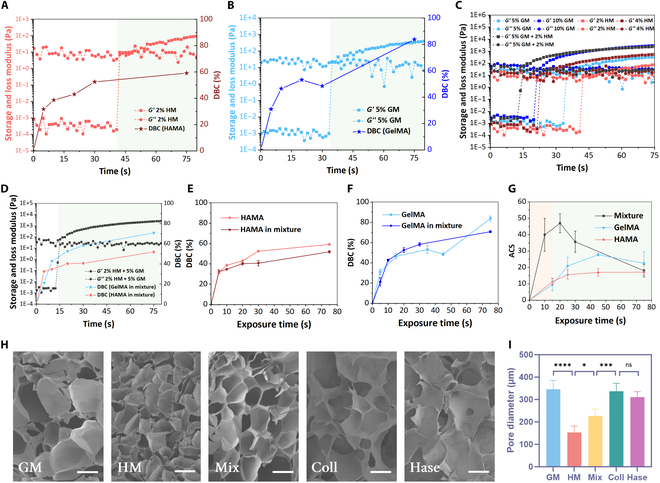
Polymerization characteristics of multi-material bioinks. Photorheological–polymerization properties of gelatin methacryloyl (GelMA) (A) and hyaluronic acid methacryloyl (HAMA) (B) single-component inks. (C) Photorheological properties of inks with different components. (D) Photo-cross-linking process of multicomponent ink. Time-varying law of DBC during separate and mixed curing of HAMA (E) and GelMA (F) hydrogels. (G) The ACS evolution of single-component and multicomponent inks during photocuring. (H) Microscopic network structure after photopolymerization of different inks. The scale bar is 200 μm. (I) Comparison of pore sizes. *Significant *P* value < 0.05; ***significant *P* value < 0.001; ****significant *P* value < 0.0001; ns, not significant. GM, GelMA; HM, HAMA; Coll, collagenase; Hase, hyaluronidase.

To minimize material interference, 2% w/v HAMA and 5% w/v GelMA with 60% degree of amino substitution (GelMA60) hydrogels were selected, which had an equal proportion of photo-cross-linkable groups (Table S1). Single-component tests revealed that the gel point (GP) for 2% HAMA was 44 s, while for 5% GelMA60, it was 34 s (Fig. 2A and B). Doubling the concentration of the single components reduced the GP time (to 22 s for 10% GelMA60 and 24 s for 4% HAMA). However, the multicomponent hydrogel composed of 5% GelMA60 + 2% HAMA, with an equal proportion of photo-cross-linkable groups, exhibited a faster GP time of 16 s (Fig. 2C), implying the “polymerization acceleration effect”. This effect may be attributed to synergistic interactions between the photosensitive groups, interpenetrating cross-linked networks, and enhanced light scattering.

To elucidate this effect, the time-evolution patterns of double bond conversion (DBC) for HAMA during the curing process of single-component and multicomponent hydrogels were tested, respectively (Fig. 2D). The results indicated no marked difference between them (Fig. 2E). A similar trend was observed for GelMA (Fig. 2F), confirming that the accelerated polymerization was not due to an increase in the absolute DBC or its growth rate. Furthermore, the dynamic average cross-linking step (ACS) test revealed that multicomponent inks exhibited a higher initial ACS (43.2 at 15 s) compared to single-component inks (9.6 for GelMA and 12.3 for HAMA at 15 s) (Fig. 2G). This suggests that during the initial curing stage, monomer molecules in multicomponent inks undergo more chain growth reactions, leading to a rapid increase in the system’s average molecular weight, subsequently increasing solution viscosity and ultimately elevating the GP. However, as exposure time increased, the ACS of multicomponent inks gradually decreased and stabilized after 80 s of exposure (full curing), with minimal difference from single-component inks. The peak ACS time for multicomponent inks aligned with the GP around 20 s, while single-component inks reached their peak near 45 s, matching the GP time (Fig. 2G). This indicates that both multicomponent and single-component inks primarily undergo chain growth reactions before the GP and termination reactions after the GP, which is closely related to the rapid increase in system viscosity after the GP, hindering molecular diffusion.

Notably, only the ACS of multicomponent inks exhibited a pronounced trend of initial increase followed by decrease, while the ACS of single-component inks remained relatively stable with minor changes. This suggests a synergistic effect between different photosensitive groups in multicomponent inks, facilitating the rapid occurrence of chain reactions. Specifically, these 2 photosensitive groups may synergistically promote the chain growth of monomer molecules during the initial exposure stage, leading to a rapid increase in ACS. However, as exposure time prolonged, the cross-linking reactions stabilized, and the differences in the roles of different photosensitive groups diminished, ultimately resulting in similar ACS between multicomponent and single-component inks.

To validate this hypothesis, the solidified hydrogels were freeze-dried and their microporous structures were analyzed using scanning electron microscopy. The results revealed distinct pore structures between 5% GelMA and 2% HAMA. However, the pore structure of the multicomponent hydrogel with a higher total concentration (5% GelMA + 2% HAMA) was not the densest, falling between that of 5% GelMA and 2% HAMA, indicating an important influence of the cross-linked network on the pore structure. As the cross-linked network of the multicomponent ink lay between those of the 2 single-component inks, its microporous structure also fell in between. Furthermore, selective degradation of GelMA and HAMA components in the multicomponent ink was conducted using collagenase and hyaluronidase (Fig. 2H), respectively. Notably, despite the degraded materials containing only HAMA or GelMA, their pore structures deviated obviously from the networks formed directly by single-component solidification. Instead, the 2 degraded materials exhibited similar pore structures (Fig. 2I). This finding further supports the higher frequency of chain propagation reactions within the mixed hydrogel during photocuring. The interpenetration of different monomers facilitated the formation of a 3D polymer network, such that selective degradation of individual components did not alter the overall network structure.

The polymerization acceleration effect implies that exposure parameters and slice images are crucial for print quality, especially at the interfaces between inks. Neglecting this effect may lead to overexposure and subsequent resolution loss. However, inadequate interfacial bonding strength can result in material domain separation. Therefore, more precise and targeted optimization and improvement of interfacial polymerization behavior are necessary.

### Material requirements of multi-material PBBP

Human tissues often exhibit a combination of hard and soft properties, such as bone, cartilage, skin, and muscle. To simulate the tissue microenvironment more accurately, multi-material 3D bioprinting often employs hydrogels with different mechanical properties to mimic various tissues. However, hydrogels with different mechanical strengths may encounter issues of mechanical mismatch and inadequate interfacial bonding strength. Therefore, investigating the interfacial bonding strength of soft–hard composite hydrogels and determining whether material combinations are suitable for multi-material 3D bioprinting are crucial for optimizing the printing process.

#### Printability curve of multi-material PBBP

Inadequate interface bonding strength can easily lead to structural separation, ultimately resulting in print failure. Therefore, establishing a standard method to determine multi-material printability is a prerequisite for successful printing. Here, we propose a criterion for evaluating multi-material printability based on interfacial strength. Due to the huge differences in inherent mechanical properties among different materials, evaluating them using a single mechanical parameter such as Young’s modulus or elongation at break cannot fully reflect the interfacial bonding behavior of the materials. Therefore, fracture energy is used as a key indicator to evaluate the interfacial bonding behavior. Fracture energy quantifies the energy absorbed by the material during uniaxial tension and is calculated as the integral area under the stress–strain curve. It comprehensively considers multiple mechanical properties, including strength, toughness, and plasticity, providing a more comprehensive reflection of the interfacial bonding behavior.

**Fig. 3.**
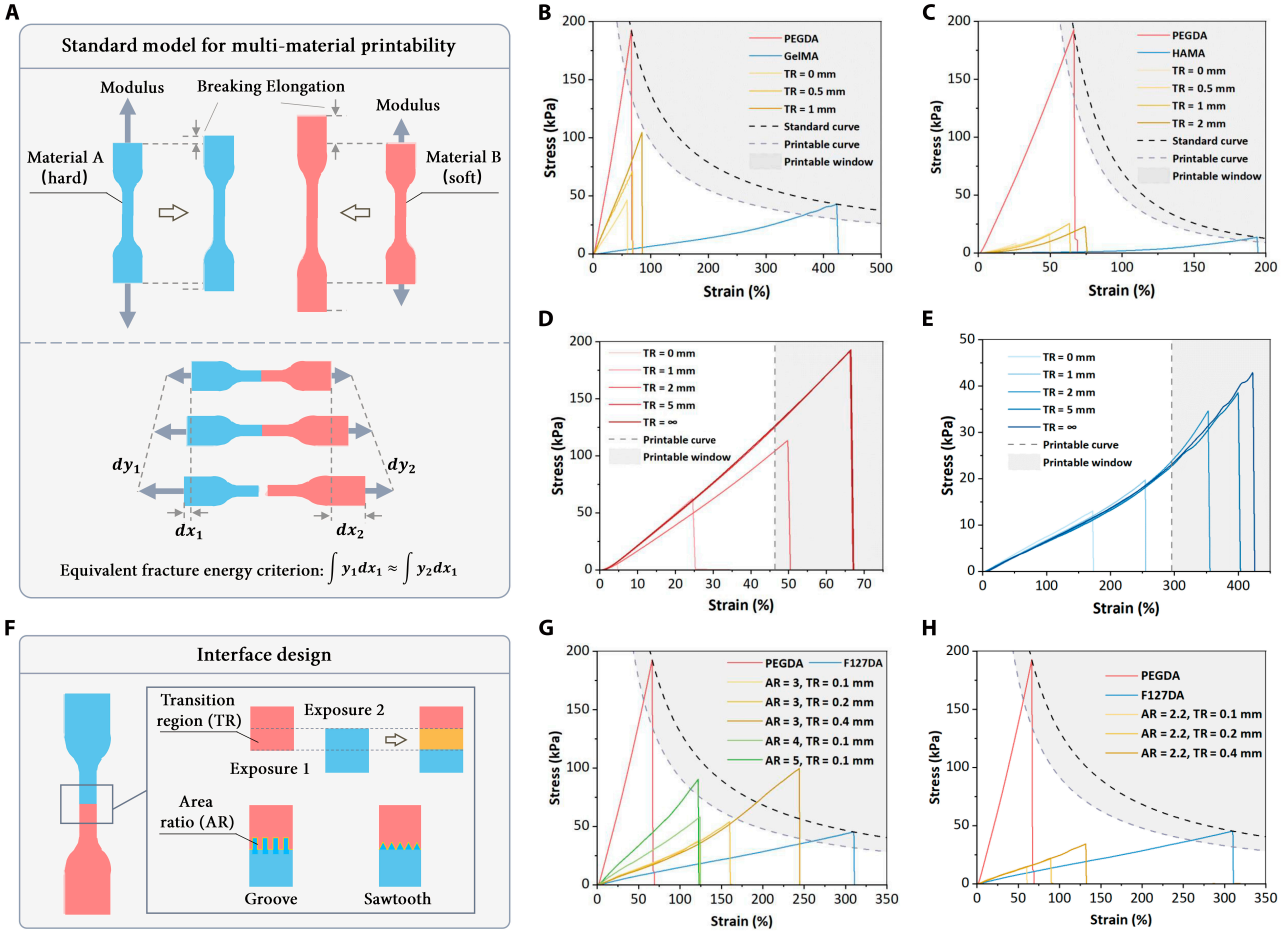
Interface strength requirements and improvements for multi-material PBBP. (A) Interfacial bonding strength requirements for mechanical property matching in multi-material PBBP. Demonstration of multi-material mechanical adaptability evaluation criteria using GelMA–poly(ethylene glycol) diacrylate (PEGDA) (B), HAMA–PEGDA (C), PEGDA–PEGDA (D), and GelMA–GelMA (E) as examples. (F) Interface design for improving bonding strength. Improvement of bonding strength by groove (G) and sawtooth (H) structures with different transition regions and area ratios.

Specifically, the standard test model is a dumbbell-shaped structure (Fig. 3A). The left and right halves are printed using 2 different inks to be tested. Subsequently, a uniaxial tensile test is conducted, and the stress–strain curve is plotted. If the fracture energy of the multicomponent ink is the same or slightly lower than that of the single-component ink, it is considered to have sufficient interfacial strength for successful multi-material printing. Firstly, this implies that the 2 materials should exhibit similar fracture energy values, as represented by the following equation:

$$\int y_{1}dx_{1}\approx \int y_{2}dx_{2}$$ (3)

Hydrogels are a class of viscoelastic materials that exhibit a linear stress–strain response within a limited range of tensile loading. In bioprinting, the hydrogels used are often ultrasoft materials [38], allowing their stress–strain behavior to be approximated as linear for practical applications. Thus, the following equal fracture energy function can be utilized within the framework of stress–strain curve analysis:

$$\frac{x\times f\left(x\right)}{2}=E$$ (4)

The equivalent fracture energy criterion translates into the following equation for a multi-material system:

$$f\left(x\right)=\frac{k}{x^{b}}\;\left(b>0\right)$$ (5)

The ideal value of b should approach 1 as closely as possible. Deviations from 1 exceeding a predefined threshold can be indicative of marked material property mismatch.

For material A, the elongation at break and tensile strength were recorded as data point $P_{1}\left(x_{1},\;y_{1}\right)$. Similarly, the data point of materials B is recorded as $P_{2}\left(x_{2},\;y_{2}\right)$. The constant in the standard fracture energy curve can be calculated using the following equation:

$$b=\text{ln}\left(\frac{y_{1}}{y_{2}}-\frac{x_{2}}{x_{1}}\right),k=y_{1}\times x_{1}^{b}$$ (6)

By relaxing this constraint appropriately, we can define a printable curve of multi-material printing (Fig. S7A):

$$p\left(x\right)=P\times \frac{k}{x^{b}}\;\left(b>0\right)$$ (7)

where P can be taken as 0.6 to 0.8.

It is noteworthy that not all interfaces encountered in multi-material printing involve dissimilar materials. A considerable portion comprises bonding interfaces between identical materials. The printable curve for such a single-material interface can be described by the following equation (Fig. S7B):

$$p\left(x\right)=P\times x_{1}$$ (8)

After establishing multi-material printability criteria, tests were conducted on bioinks: PEGDA, GelMA, HAMA, and F127DA. The material combinations tested included PEGDA–GelMA, PEGDA–HAMA, PEGDA–F127DA, PEGDA–PEGDA, and GelMA–GelMA. In the PEGDA–GelMA combination, PEGDA exhibited high strength (191 kPa) but low elongation (64%), appearing hard and brittle; GelMA had low strength (52 kPa) but high elongation (425%), appearing soft and tough. Results indicated low interfacial strength for PEGDA–GelMA, not meeting the printable window (Fig. 3B). Similar findings were observed for PEGDA–F127DA (Fig. S8) and PEGDA–HAMA (Fig. 3C). F127DA’s strength was close to that of GelMA, while that of HAMA was lower (19 kPa). Both failed to meet the printable window with PEGDA. Single-material interfaces showed similar results, with PEGDA–PEGDA (Fig. 3D) much lower and GelMA–GelMA slightly higher but still not reaching the printable window (Fig. 3E). GelMA–GelMA’s higher strength might be due to its lower density molecular network after cross-linking, allowing monomer diffusion, enhancing strength. PEGDA’s denser network hindered monomer penetration, resulting in lower strength. However, prevalent interfacial mechanical mismatch leading to insufficient strength indicates that, without optimization, multi-material 3D bioprinting risks material separation, affecting printing quality, especially for structures that need to bear loads.

#### Interface structure design

Optimizing interface structure design enhances multi-material 3D bioprinting printability. We use the transition region (TR) and area ratio (AR) to characterize the interface (Fig. 3F). TR is the overlap length during exposures, and AR is the ratio of interface contact to the cross-sectional area. Varying TR values revealed that PEGDA–GelMA and PEGDA–F127DA achieved a printable window at TR = 1 mm, addressing interfacial mechanical mismatch (Fig. 3B and Fig. S8). However, PEGDA–HAMA remained far from the printable window at TR = 2 mm (Fig. 3C), indicating excessive mechanical differences unsuitable for multi-material printing via interface design. Notably, multi-material interfaces reached the printable window at TR = 1 mm, while single-material PEGDA–PEGDA (Fig. 3D) and GelMA–GelMA (Fig. 3E) required TR = 1.9 mm and TR = 1.4 mm, respectively. This supports the polymerization acceleration effect in multicomponent bioinks, leading to faster cross-linking and smaller TR values for printability.

To assess AR’s impact, groove and sawtooth structures with varying TRs and ARs were tested using PEGDA–F127DA. For both structures, increasing TR at a constant AR boosted interface strength and elongation (Fig. 3G and H). Conversely, increasing AR at a constant TR enhanced strength but not elongation. This is because a larger TR creates thicker interfaces, resisting deformation, while a larger AR reduces stress per unit area, increasing strength. For specific applications, AR and TR should be tailored based on the desired mechanical properties.

### Cross-contamination in multi-material PBBP

Cross-contamination is a great challenge in multi-material PBBP, severely affecting the quality and performance of printed structures. It is manifested in the following 2 aspects: (a) infiltration contamination of hydrogel polymerization networks due to a high water content and frequent ink switching and (b) residual contamination during ink switching, where inadequate cleaning can lead to contamination and altered ink properties.

#### Infiltration contamination

**Fig. 4.**
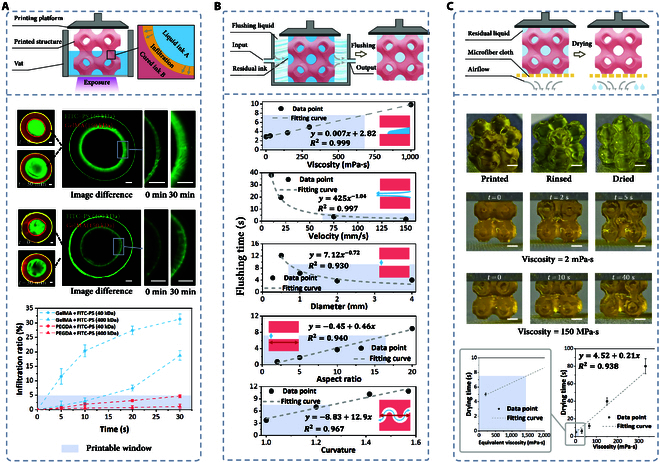
Cross-contamination issues and solutions in multi-material PBBP. (A) Infiltration contamination analysis and printable window in multi-material printing processes The scale bar is 500 μm. (B) The impact of rinsing parameters and structural parameters on rinsing efficiency, and the determination of the printable window. (C) Drying process of negative-pressure-assisted capillary adsorption and determination of the printable window. The scale bar is 2 mm. FITC–PS, fluorescein isothiocyanate–polysucrose.

The high water content of hydrogels facilitates the pronounced diffusion of hydrophilic molecules, while the extended printing time (typically several hours) for multi-material PBBP makes infiltration contamination a common occurrence (Fig. 4A). To investigate this phenomenon, a standard protocol employing an annular structure with a substrate as a model for infiltration was established. Under fluorescence microscopy, the diffusion of fluorescent liquids, such as fluorescein isothiocyanate (FITC)–dextran, into the hydrogel was observed.

Viscous bioink penetration can be categorized into 4 types: small molecules into high-density-of-molecular-network (DMN) hydrogels, small molecules into low-DMN hydrogels, large molecules into high-DMN hydrogels, and large molecules into low-DMN hydrogels. PEGDA (700 Da) served as the high-DMN hydrogel (DMN = 10^0^ to 10^1^ nm^−2^), GelMA60 (150 kDa) as the low-DMN hydrogel (DMN = 10^−1^ to 10^0^ nm^−2^), FITC–polysucrose (PS) (40 kDa) as the small molecule, and FITC–PS (400 kDa) as the large molecule. Results showed that after 30 min, the permeability of 40 kDa FITC–PS in GelMA was 31.2%, while 400-kDa FITC–PS’s permeability was 18.7%. Similarly, permeabilities in PEGDA were 4.69% and 1.12% (Fig. S9) for 40 and 400 kDa, respectively. These findings suggest a negative correlation between DMN and permeability, as well as between molecular weight and permeability. Considering a 5% permeability threshold as the printable window, permeation contamination is almost inevitable in low-DMN hydrogels, whereas it is obviously reduced in high-DMN hydrogels. Therefore, when printing low-DMN hydrogels, the accompanying material should have a larger molecular weight to mitigate permeation contamination to some extent. Conversely, combinations of materials with large disparities in molecular weights will result in more pronounced effects.

High-viscosity material combinations or thickening agents have potential practicality in mitigating infiltration contamination. Silk fibroin [39] and hyaluronic acid [40] can be used to enhance bioink viscosity, preventing cell sedimentation and reducing infiltration in multi-material PBBP. Another point to consider is that small molecular dyes, such as tartrazine and Sudan Red, are commonly used as photoabsorbers in PBP to control polymerization reactions and enhance printing resolution. However, these small-molecule dyes can also cause permeation contamination, thereby altering the photoresponsive properties of the ink. A viable solution is to use macromolecular mineral dyes. These dyes primarily control light scattering and transmission through particle shading, so the impact of color on absorbance is relatively minor. Additionally, they can effectively prevent cross-contamination (Fig. S11).

#### Residual contamination

A suitable cleaning procedure can effectively prevent residual contamination. However, traditional methods such as high-pressure airflow and high-speed centrifugation are not suitable for ultrasoft bioinks due to potential structural damage, necessitating gentler approaches. We developed a fluidic controlled rinsing method. Additionally, porous structures commonly found in medical applications, such as multilayer vascular networks and porous scaffolds, pose great challenges to cleaning efficiency. These structures provide hiding spaces for bioinks, making it crucial to effectively flush bioinks from porous structures while avoiding structural damage. Moreover, the inherent hydrophilicity of hydrogels makes them highly susceptible to water absorption, thus necessitating a drying step after cleaning to prevent swelling of the printed structure and changes in bioink concentration.

In the multi-material PBBP process, there are hundreds of cleaning operations, so optimizing cleaning efficiency is crucial for improving overall efficiency. The key parameters affecting cleaning efficiency can be divided into 2 categories: fluid parameters and structural parameters. Fluid parameters include the velocity of the rinsing liquid and the viscosity of the bioink, while structural parameters include the diameter, depth-to-width ratio, and curvature of channels. The influence of these parameters on rinsing efficiency was investigated using computational fluid dynamics simulations. Clean rinsing was defined as the residual ink within the channels being less than 1% (Fig. S12). To ensure that printing time remains reasonable, a rinsing time of less than 7.5 s was chosen as the printable window.

For fluid parameters, ink viscosity is directly related to rinsing time, and the ink viscosity should not exceed 700 mPa·s. Rinsing velocity is inversely proportional to rinsing time, with a velocity greater than 60 mm/s performing well, but it should not exceed 150 mm/s to avoid structural deformation. As for structural parameters, channel diameter directly affects rinsing efficiency, especially in extremely fine pores with diameters below 0.8 mm. Both aspect ratio and curvature are positively correlated with rinsing time, with curvature having a more pronounced effect; the aspect ratio should not exceed 17, and curvature should ideally be below 1.25 (Fig. 4B).

After fluid rinsing, drying the printed structures is essential. Residual rinsing fluid can dilute the bioink, leading to printing failures. We propose a negative-pressure-assisted capillary adsorption method, which effectively removes residual rinsing fluid through the capillary effect of ultrafine fiber cloths while protecting the printed structures. Experiments demonstrate that residual fluid in porous 3-periodic minimal surface structures can be effectively removed within a short time. It should be noted that the rinsing process also has a substantial impact on the drying process. Inadequate rinsing can lead to increased viscosity of the residual liquid, prolonging the drying time (Movie S3). For instance, with adequate rinsing (residual liquid viscosity of 2 mPa·s), a drying time of 5 s is sufficient. Conversely, insufficient rinsing (residual liquid viscosity of 100 mPa·s) requires over 40 s. Therefore, with a drying time of 7.5 s as the printable window, the equivalent material viscosity should be below 1,400 mPa·s (Fig. 4C). Moreover, it is worth noting that the purpose of this process is to remove residual cleaning solution from the surface of the printed structure. This process, typically completed within 7.5 s, does not affect the internal environment of the hydrogel and therefore has a negligible impact on cell viability.

### Printing resolution of multi-material PBBP

Resolution is a key metric for assessing 3D-printing capabilities, but current research on print resolution mainly focuses on single-material printing [14,15], with little systematic study on multi-material PBBP. Multi-material printing complicates the process due to the variety of materials with different characteristics, making error source analysis and evaluation criterion development challenging. In multi-material PBBP, printing errors primarily stem from 3 sources.

#### Type A resolution degradation

Type A resolution degradation represents dimensional deviations caused by insufficient or excessive photocuring. Insufficient photocuring leads to low structural strength, prone to collapse and deformation. Excessive photocuring results in increased print structure dimensions. The corresponding resolution is denoted as $R_{\text{first}}$.

#### Type B resolution degradation

Type B resolution degradation represents mutual influence of intralayer multi-material printing. The cured structure occupies space, restricting the flow and filling of subsequent materials, leading to uneven dimensions. Additionally, differences in the refractive index of different materials cause light refraction, affecting the exposure of subsequent materials and resulting in inconsistent dimensions. The corresponding resolution is denoted as $R_{\text{second}}$.

#### Type C resolution degradation

Type C resolution degradation represents resolution reduction due to ink cross-contamination. The mixing of different inks during printing alters material properties, affecting photocuring effectiveness and reducing resolution. Alternatively, residual ink from the previous material in the printing area affects the adhesion and curing of subsequent inks, lowering resolution. The corresponding resolution is denoted as $R_{\text{cross}}$.

#### Printing resolution standard test model

**Fig. 5.**
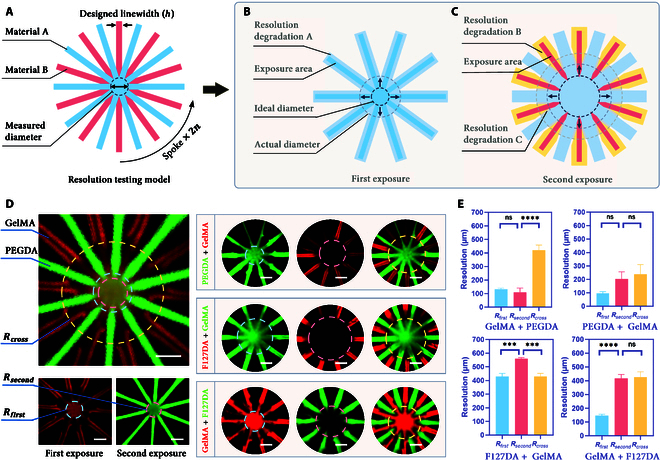
Resolution evaluation and analysis of multi-material PBBP. (A) Schematic of the resolution evaluation model; (B) intralayer printing resolution degradation caused by overexposure; (C) interlayer printing resolution degradation caused by overexposure and resolution degradation caused by material infiltration; (D) analysis of multi-material printing resolution using F127DA–GelMA, GelMA–F127DA, PEGDA–GelMA, and GelMA–PEGDA. The scale bar is 100 μm. (E) Comparison of printing resolutions for different materials and exposure sequences. ***Significant *P* value < 0.001; ****significant *P* value < 0.0001; ns, not significant.

To quantitatively print resolution, we designed a standard test model with alternating spoke structures (Fig. 5A). This model was printed using an intralayer multi-material method, involving 2 exposure steps. In the first step, ink A was cured to form *n* spoke structures (Fig. 5B). The vat was then switched to ink B, and the exposure pattern was rotated to create another *n* spoke structures, resulting in a total of 2*n* spokes (Fig. 5C). This spoke structure enables the measurement of both lateral and axial resolutions by analyzing the diameter of the circular spokes. The resolution calculation method is as follows:

$$R=D\times \text{tan}\left(\frac{\pi }{n}\right)-\frac{h}{\text{cos}\left(\frac{\pi }{n}\right)}$$ (9)

where D is the diameter of the central circular area, h is the width of each spoke, and n is the number of spokes.

To evaluate the performance of the multi-material print resolution test model, 4 common ink combinations (GelMA–PEGDA, PEGDA–GelMA, F127DA–GelMA, and GelMA–F127DA) were examined. Fluorescent dyes were covalently bonded to the hydrogel network to prevent diffusion and aid visualization. The diameters of the central circles were measured after the first and second exposures to calculate $R_{\text{first}}$, $R_{\text{second}}$, and $R_{\text{cross}}$, respectively.

The results indicated that the $R_{\text{first}}$ and $R_{\text{second}}$ of GelMA–PEGDA were 133 and 110 μm, respectively. This was because the DMN of GelMA was relatively low, allowing small molecules PEGDA to penetrate, resulting in $R_{\text{second}}$ being lower than $R_{\text{first}}$. However, at the same time, this penetration also led to a marked increase in $R_{\text{cross}}$ (421 μm). Interestingly, when the exposure order was reversed to PEGDA–GelMA, $R_{\text{first}}$ and $R_{\text{second}}$ were 95 and 202 μm, respectively. This was due to the higher DMN of PEGDA cured first, which restricted the penetration of large GelMA molecules, leading to an obvious increase in $R_{\text{second}}$. Correspondingly, the lower permeability also reduced $R_{\text{cross}}$ to 237 μm (Fig. 5D and E).

In the F127DA–GelMA group, $R_{\text{first}}$ and $R_{\text{second}}$ were 428 and 560 μm, respectively. F127DA itself had poor formability, resulting in a higher $R_{\text{first}}$. Although GelMA had better formability than F127DA, $R_{\text{second}}$ was also affected by type B resolution degradation due to F127DA being cured first. It is worth noting that $R_{\text{cross}}$ was basically consistent with $R_{\text{first}}$, indicating that the type A resolution degradation of F127DA overshadowed type C resolution degradation, making it difficult to distinguish between the 2. When the exposure order was changed to GelMA–F127DA, $R_{\text{first}}$ and $R_{\text{second}}$ were 146 and 415 μm, respectively. The substantial reduction in $R_{\text{first}}$ demonstrated the profound impact of exposure order on print resolution. The results indicate that exposing high-DMN hydrogels before low-DMN hydrogels enhances $R_{\text{first}}$ and $R_{\text{cross}}$ but is detrimental to $R_{\text{second}}$, and vice versa. These results emphasize the importance of optimizing the printing process based on the specific characteristics of material combinations, rather than merely combining materials in isolation. The exposure order is a critical yet often overlooked factor (Fig. 5D and E).

### Characterization of multi-material PBBP

**Fig. 6.**
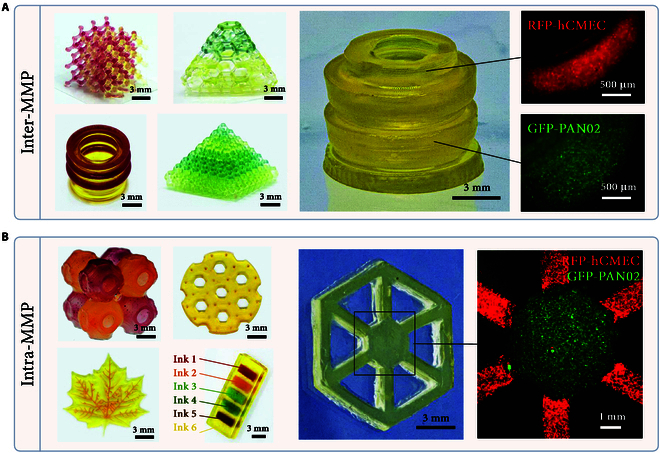
Various lattice structures, 3-periodic minimal surface (TPMS) (A), drug delivery, cell-laden biomimetic islets, and trachea models (B) printed by multi-material PBBP.

To demonstrate the printing quality of Inter- and Intra-MMP, a series of experiments were conducted. For Inter-MMP, lattice structures with various interweaving, nesting, and overhanging features were successfully fabricated. The printed structures exhibited sharp material interfaces, high structural fidelity, and desirable mechanical properties. Additionally, a biomimetic tracheal model was designed and printed with 2 types of cell-laden bioinks (RFP-hCMEC and GFP-PAN02) (Fig. 6A). The distinct cell populations within different layers of the model highlight the effective avoidance of cross-contamination.

For Intra-MMP, 3-periodic minimal surface and drug-releasing chip models were fabricated. Despite the increased complexity of ink switching and cleaning steps, clear material interfaces and high printing resolution were achieved by optimizing printing parameters. Furthermore, a liver lobule model was constructed with 2 types of cell-laden bioinks (RFP-hCMEC and GFP-PAN02) (Fig. 6B). The distinct cell populations in the center and periphery of the model demonstrate the precise control over material deposition.

These results collectively underscore the significance of investigating the printability of multi-material PBBP in constructing complex biological structures with high fidelity and efficiency. This technology holds great promise for advancing biomedical research and tissue engineering applications.

## Discussion

In this study, we systematically investigated the key principles and process requirements underlying multi-material PBBP. The primary limitations in this field have been identified as the dearth of systematic research and the lack of mature printing equipment. To address these challenges, we conducted a comprehensive exploration of multi-material PBBP, encompassing ink switching, polymerization kinetics, material compatibility, cross-contamination, printing resolution, and equipment optimization. We demonstrated the feasibility of our approach using a variety of commonly employed bioinks. Through this work, we aim to provide researchers with essential insights to facilitate the widespread adoption and practical application of multi-material PBBP technologies.

Despite effective advancements in optimization strategies, multi-material PBBP, particularly intra-material printing, remains a time-consuming process. This extended printing time can adversely impact cell viability and biocompatibility, especially in cell-laden printing applications. Addressing this limitation may require the development of innovative technologies. Additionally, the diversity of bioink types presents further challenges. While this study focuses on free-radical-induced photo-cross-linking hydrogels, exploring alternative polymerization mechanisms and bioink formulations is crucial for expanding the application scope of multi-material PBBP.

## Materials and Methods

### Test of photorheological properties

The rheological properties of hydrogel solutions were tested with a rheometer (MCR302, Anton Paar, Austria) equipped with a 405-nm light source. The exposure intensity was set to 30 mW/cm^2^; 0.25% w/v lithium phenyl-2,4,6-trimethylbenzoylphosphinate (LAP) (EFL-Tech, China) and 0.05% w/v lemon yellow (EFL-Tech, China) were added into 10% w/v hydrogel solution. The viscosities of the hydrogel solutions were tested with a steady-rate sweep and a shear rate range from 0.01 to 1,000 s^−1^. A conical plate with a diameter of 40 mm and a truncation gap distance of 100 μm was used in this work. All rheological tests were performed at 37 °C.

### Calculation of DBC

#### Measurement of nuclear magnetic resonance

GelMA (EFL-Tech, China), HAMA (EFL-Tech, China), collagenase II (EFL-Tech, China), and hyaluronidase (Aladdin, China) solutions were prepared with $D_{2}O$ (Aladdin, China). The cross-linked GelMA and HAMA were completely degraded into liquid by collagenase II and hyaluronidase solutions, respectively, and then diluted to 1% concentration with $D_{2}O$; 0.5 ml of the diluted solution was pipetted and added to a nuclear magnetic resonance (NMR) tube, and the ^1^H spectrum was measured by an NMR spectrometer (AVANCE III, Bruker company, Switzerland).

#### Calculation of DBC

The DBC of cross-linked hydrogels can be calculated as follows:

DBC=1−AafterAbefore×100% (10)

where $A_{\left(\text{before}\right)}$ is the integral area of characteristic peaks of the carbon–carbon double bond in NMR spectrum before cross-linking and $A_{\left(\text{after}\right)}$ is the integral area after cross-linking.

### Calculation of ACS

#### Measurement of the P element content

Because hydrogels were often cured by excessive photoinitiators to ensure complete curing, it is necessary to eliminate excess initiators; 250 μl of 10% w/v hydrogel solution was photocured and then placed in 90% ethanol solution at 50 °C. After soaking for 60 min, the cured hydrogel was taken out. The new ethanol solution was replaced, and the above operation was repeated 3 times. The hydrogel was put into a beaker, and 1 ml of 37% nitric acid was added. Then, the beaker was heated to 100 to 120 °C for 15 min to ensure that the solid was completely digested. Then, the digested solution was diluted to 7 ml with purified water. Finally, the diluted solution was filtered through a 0.22-μm filter (Millex-GP, Merck Millipore, Ireland). The content of the P element was tested by inductively coupled plasma mass spectrometry (7800, Agilent, Singapore).

#### Calculation of ACS

The ACS is calculated as follows:

$$\mathrm{ACS}=2\times \frac{\frac{x\times \mathrm{DBC}\times \mathrm{DoF}}{M_{\mathrm{ave}}}\times 10^{-3}\times N_{A}}{\frac{2V\times \rho_{\left(P\right)}}{M_{P}}\times 10^{-9}\times N_{A}}-1=\frac{x\times \mathrm{DBC}\times \mathrm{DoF}\times M_{P}}{V\times \rho_{\left(P\right)}\times M_{\mathrm{ave}}}\times 10^{6}-1$$ (11)

where x (mg) is the mass of the hydrogel after curing, $M_{P}$ is the molecular weight of the P element, V (ml) is the volume of the solution after nitric acid digestion and dilution, $\rho_{\left(P\right)}$ (μg/l) is the P element concentration, DoF is the degree of functionalization, and $M_{\mathrm{ave}}$ is the average molecular weight of the hydrogel.

### Scanning electron microscopy

To minimize the influence of external factors on pore size, all samples were prepared from the same batch and the processing temperature was strictly controlled. After solidification, the hydrogels were pre-cooled in a −80 °C refrigerator for 2 h until completely frozen. Then, they were freeze-dried in a freeze dryer (Scientz-18ND, Japan) for 48 h until completely dry. To minimize the influence of ambient temperature, all samples were placed in the same area. Afterward, the samples were cut and their micropore structure was observed using scanning electron microscopy (Hitachi SU8010, Japan).

### Mechanical test

Dumbbell-shaped tensile test samples were printed. The thickness, width, and length of the tensile part were 1, 2, and 12 mm, respectively. The samples were tested with a 20-N force sensor on a universal testing machine (UTM2102, Shenzhen Suns Technology Stock Co., Ltd, Shenzhen, China) at a constant tensile rate of 10 mm/min, and the data were drawn as a tensile stress–strain curve.

### Infiltration pollution test

A ring-shaped structure with an outer diameter of 5 mm and an inner diameter of 3 mm was printed for the infiltration pollution test. It is worth noting that the ring structure contained a base plate (Fig. S9) to prevent the fluorescent liquid from permeating from the bottom. After printing, 5 μl of FITC–PS (Aladdin, China) solution was dropped onto the center of the structure, and it was placed under a fluorescence microscope (Guangzhou Micro-shot Technology Co., Ltd, MF53-N, China) for observation. The environmental humidity is controlled at 40% to 60%.

### Liquid rinsing efficiency simulation

The ANSYS Fluent software was employed for fluid simulation analysis. The standard *k*–*ε* model was selected as the turbulence model, coupled with the species transport model. Boundary conditions included a specified inlet velocity on one side, an outlet on the opposite side, and rigid walls on the remaining sides. The simulated fluid was assumed to be an incompressible ideal fluid, and the influence of the fluid on the printed structure was neglected. The key parameters set were velocity, viscosity, diameter, aspect ratio, and curvature. The cleaning criterion was defined as an integral bioink mass fraction within the channels of less than 1% (Fig. S12).

### Three-dimensional bioprinting

A bioink composed of 10% w/v PEGDA and 0.25% w/v LAP was prepared using phosphate-buffered saline (Aladdin, China) as the solvent. Different dyes (Fig. S11) were selected as photoabsorbers to demonstrate the multi-material printing process. Using our self-developed multi-material printer, the exposure intensity was set to 30 mW/cm^2^, the exposure time was set to 10 s, the washing time was set to 3 s, and the slicing thickness was set to 100 μm.

For cell-laden printing, red-fluorescent-protein-labeled immortalized human brain microvascular endothelial cells (RFP-hCMEC) (Shanghai Zhong Qiao Xin Zhou Biotechnology Co., Ltd., China) and green fluorescent protein-labeled mouse pancreatic cancer cells (GFP-PAN02) (Shanghai Zhong Qiao Xin Zhou Biotechnology Co., Ltd., China) were used for demonstration. After digesting the cells from the culture flask, they were washed 3 times with phosphate-buffered saline and centrifuged. An appropriate amount of the above bioink was added to adjust the cell concentration to 5 million cells/ml, and then the cells were printed in the sterile area of the multi-material printer.

## Data Availability

All data supporting the findings of this study are available in the paper and in the Supplementary Materials.
