# Supplementary material for: Printability in Multi-material Projection-Based 3-Dimensional Bioprinting
Source: Research (Wash D C). 2025 Mar 4;8:0613. doi: 10.34133/research.0613 (PMC11876545; doi:10.34133/research.0613)
Supplement: Supplementary 1 — Figs. S1 to S12 Table S1 Movies S1 to S3 [file research.0613.f1.zip › Supplymentary material.docx]

**Supplementary Materials**

**Printability in Multi-material Projection-based 3D Bioprinting**

Chao-fan He^1,2^, Tian-hong Qiao^1,2^, Xu-chao Ren^3^, Mingjun Xie^1,2^, Qing Gao^4^, Chao-qi Xie^4^, Peng Wang^4^, Yuan Sun^1,2^, Huayong Yang^1,*^, Yong He^1,2,5,6,*^

^1^State Key Laboratory of Fluid Power and Mechatronic Systems, School of Mechanical Engineering, Zhejiang University, Hangzhou 310027, China. ^2^Key Laboratory of 3D Printing Process and Equipment of Zhejiang Province, College of Mechanical Engineering, Zhejiang University, Hangzhou 310027, China. ^3^School of Computer Science, Xi‘an Shiyou University, Xi‘an 710065, China. ^4^EFL-tech, Suzhou Yongqinquan Intelligent Equipment Co., Ltd, Suzhou 215101, China. ^5^Liangzhu Laboratory, Zhejiang University, 1369 West Wenyi Road, Hangzhou 311121, China. ^6^The Second Affiliated Hospital of Zhejiang University and State Key Laboratory of Fluid Power and Mechatronic Systems, Zhejiang University, Hangzhou 310027, China.

*Address correspondence to: [yhy@zju.edu.cn](mailto:yhy@zju.edu.cn) (H.-Y.Y.); [yongqin@zju.edu.cn](mailto:yongqin@zju.edu.cn) (Y.H.).


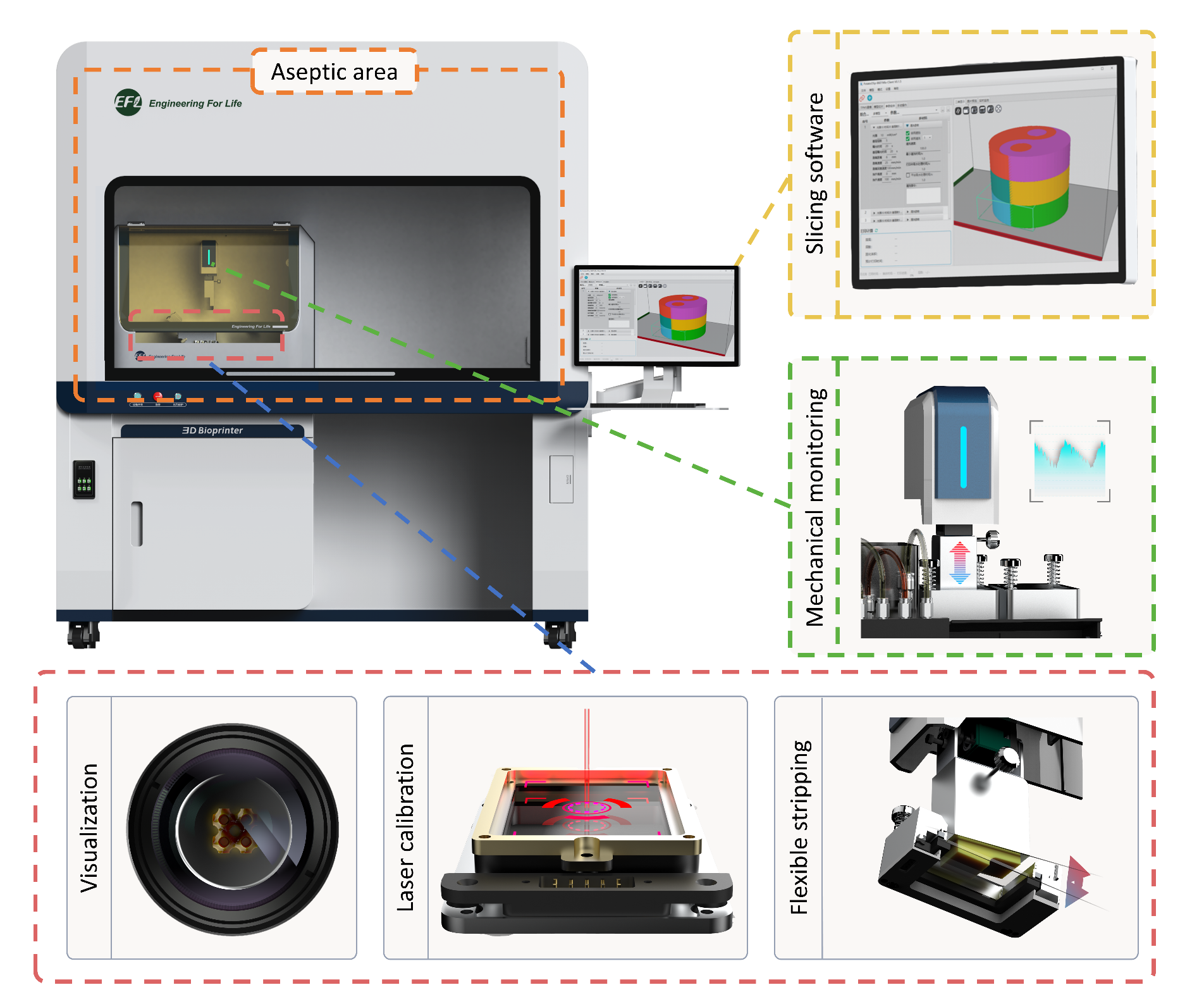


**Fig. S1**| Configuration of multi-material projection-based 3D bioprinter.


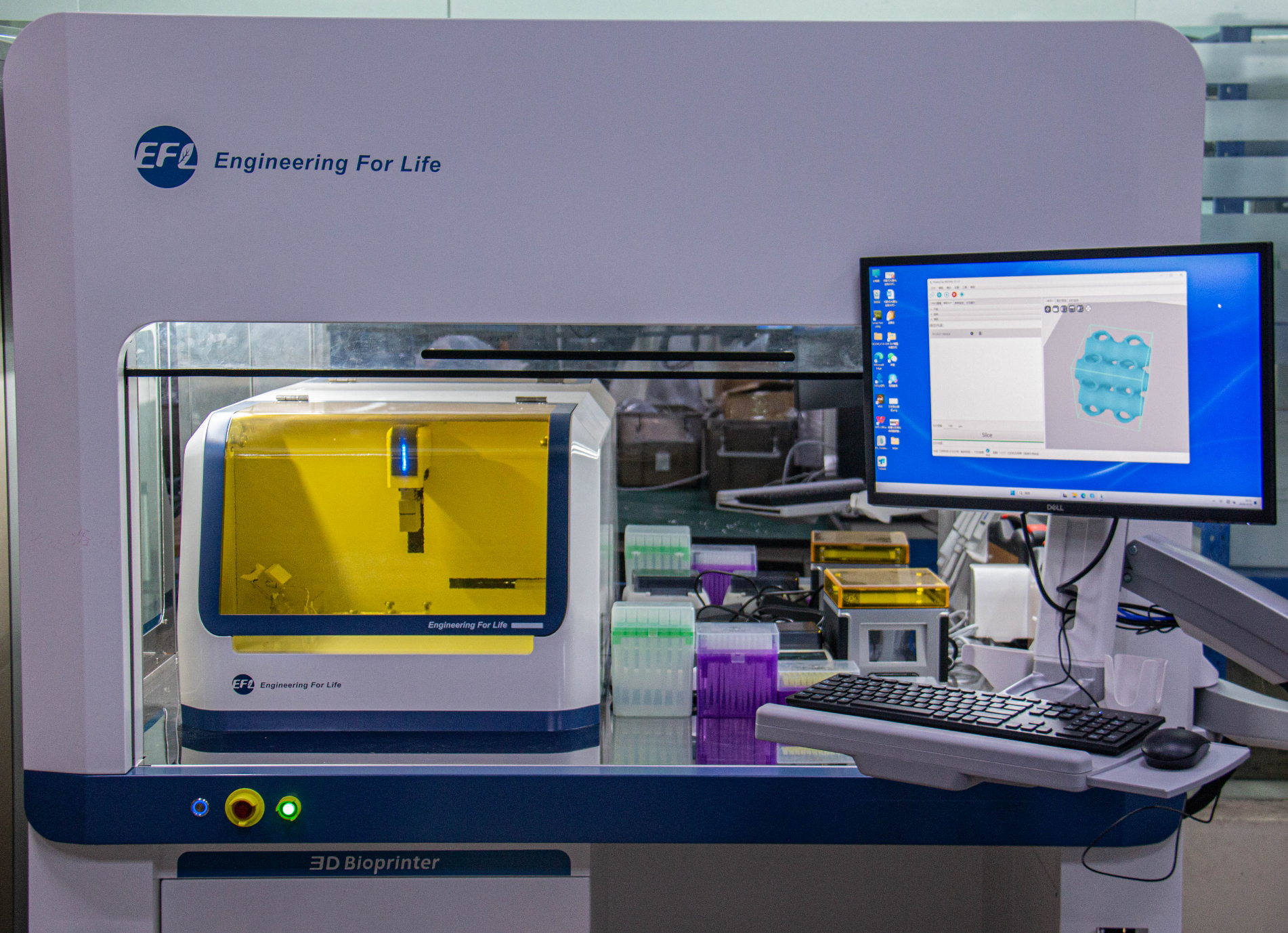


**Fig. S2**| A physical image of the 3D bioprinter.


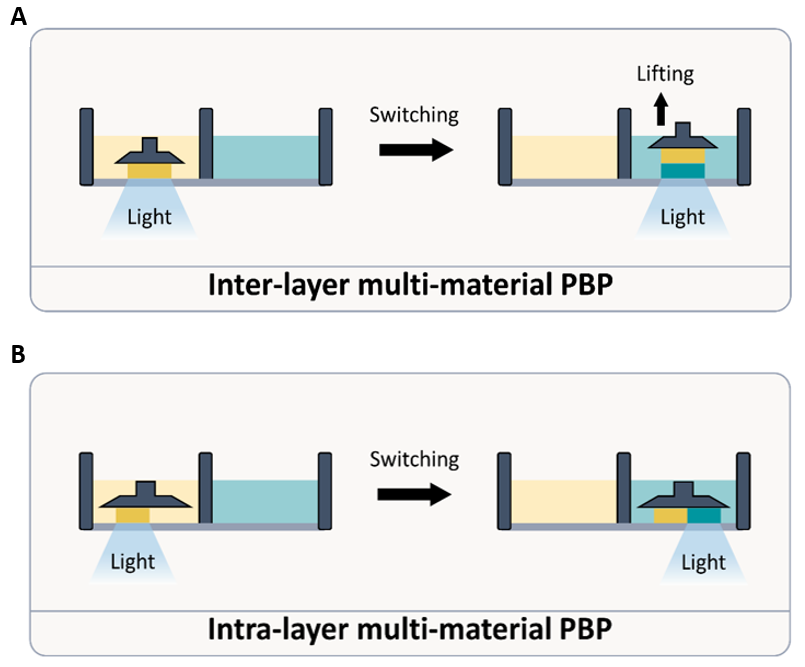


**Fig. S3**| Classification of multi-material PBP. (A) Inter-layer multi-material PBP. (B) Intra-layer multi-material PBP.


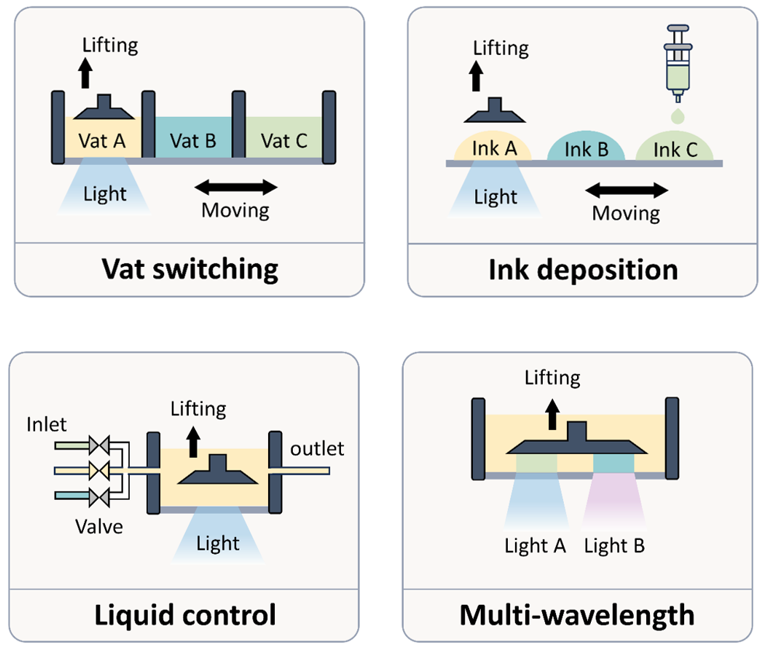


**Fig. S4**| Ink switch approaches of multi-material PBP.


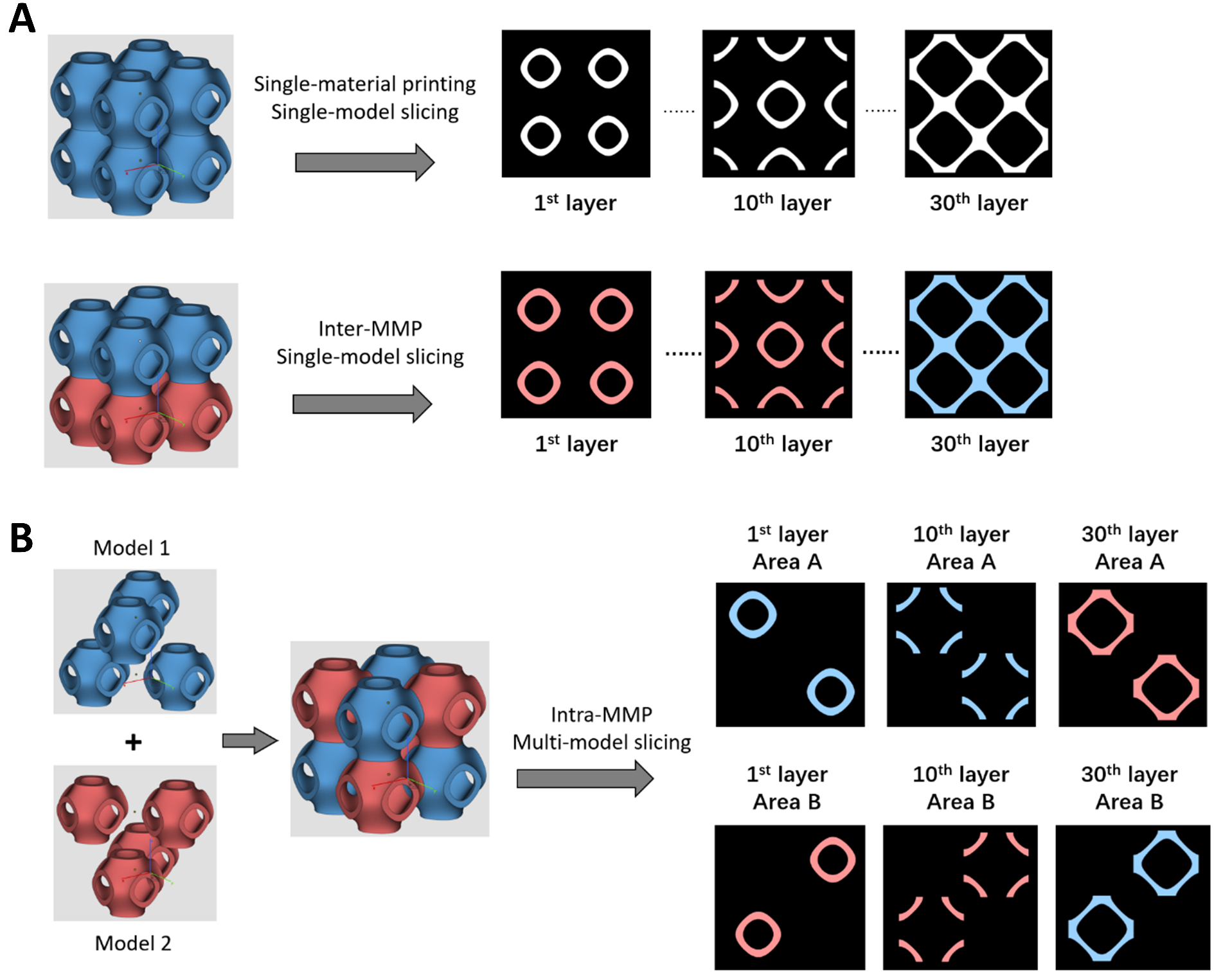


**Fig. S5**| Slicing method of Inter-MMP (A) and Intra-MMP (B).


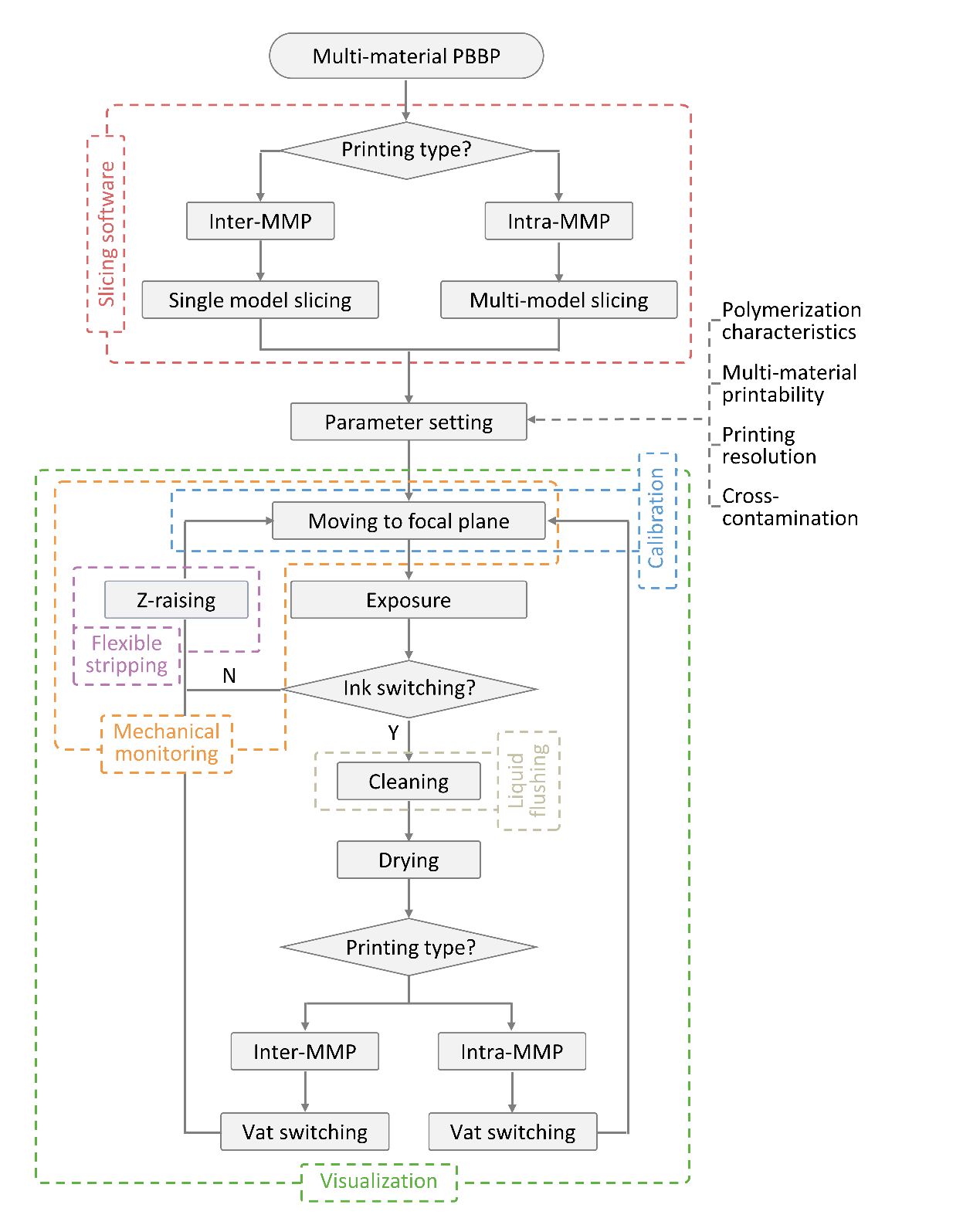


**Fig. S6**| System block diagram of the multi-material 3D bioprinter.

**
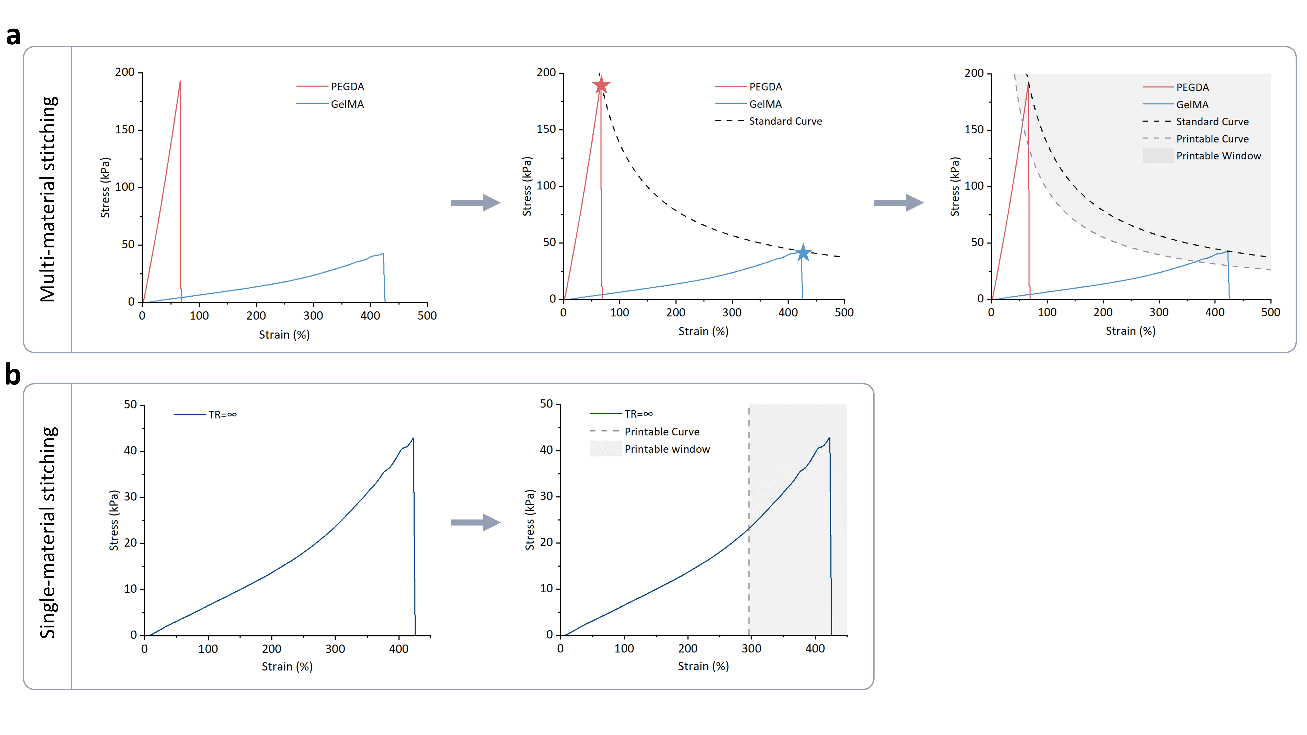
**

**Fig. S7**| Multi-material printable curve. (A) Multi-material stitching. (B) Single-material stitching.

**
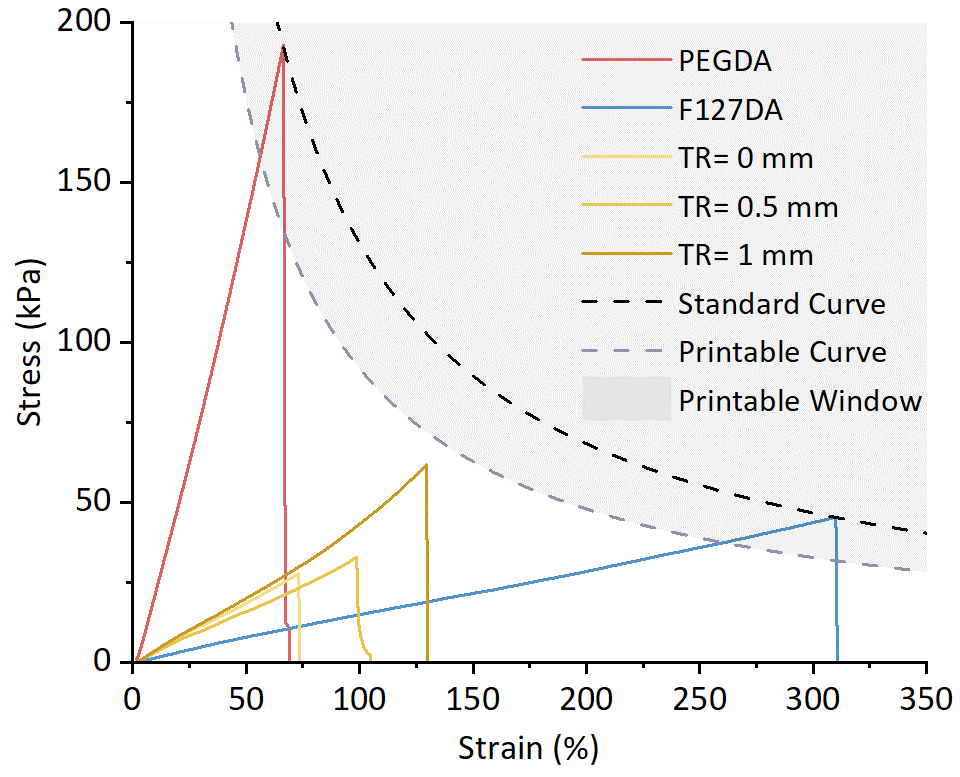
**

**Fig. S8|** Multi-material printable curve of PEGDA-F127DA.

**
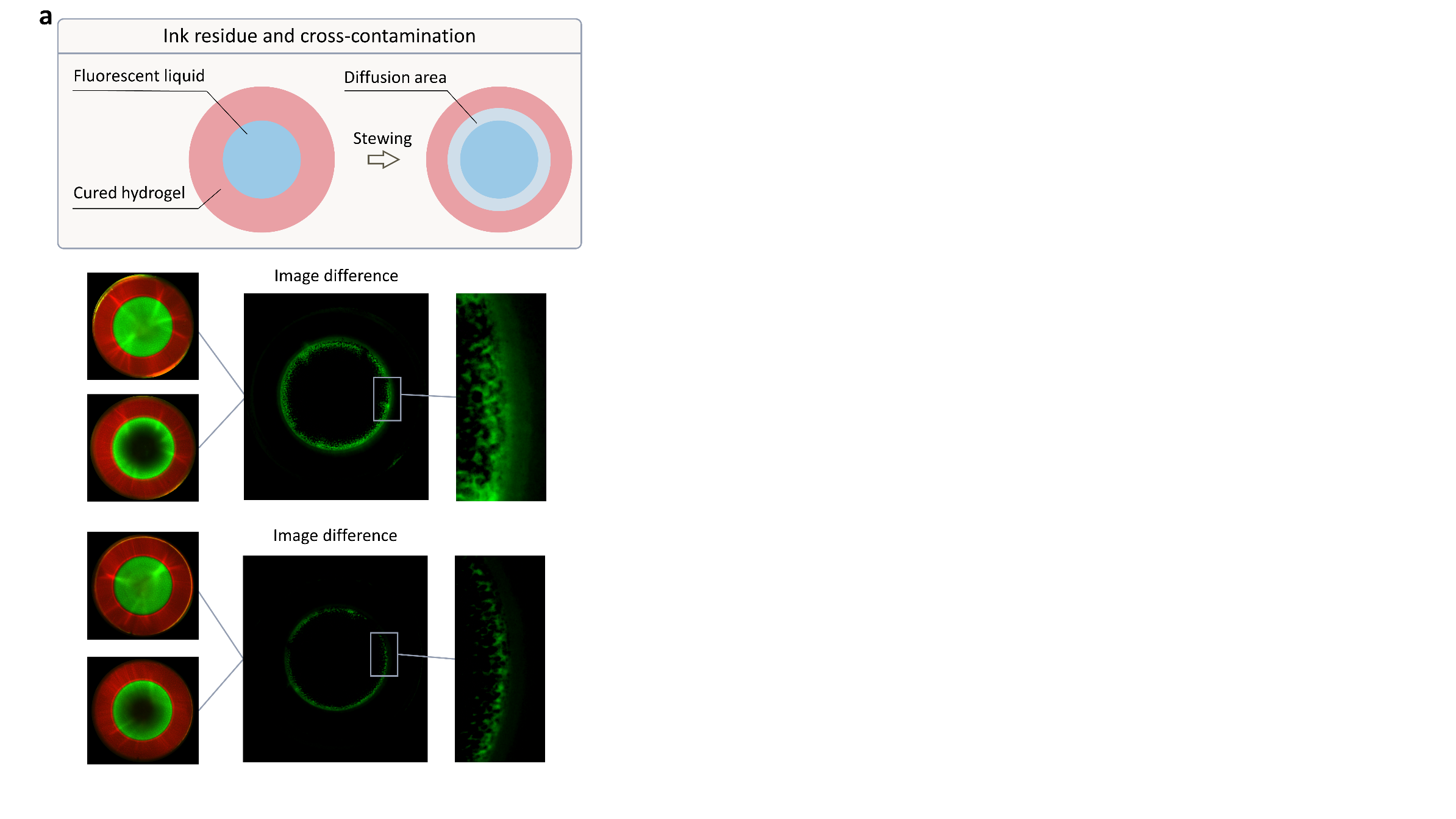
**

**Fig. S9|** Infiltration pollution test of FITC-PS to PEGDA


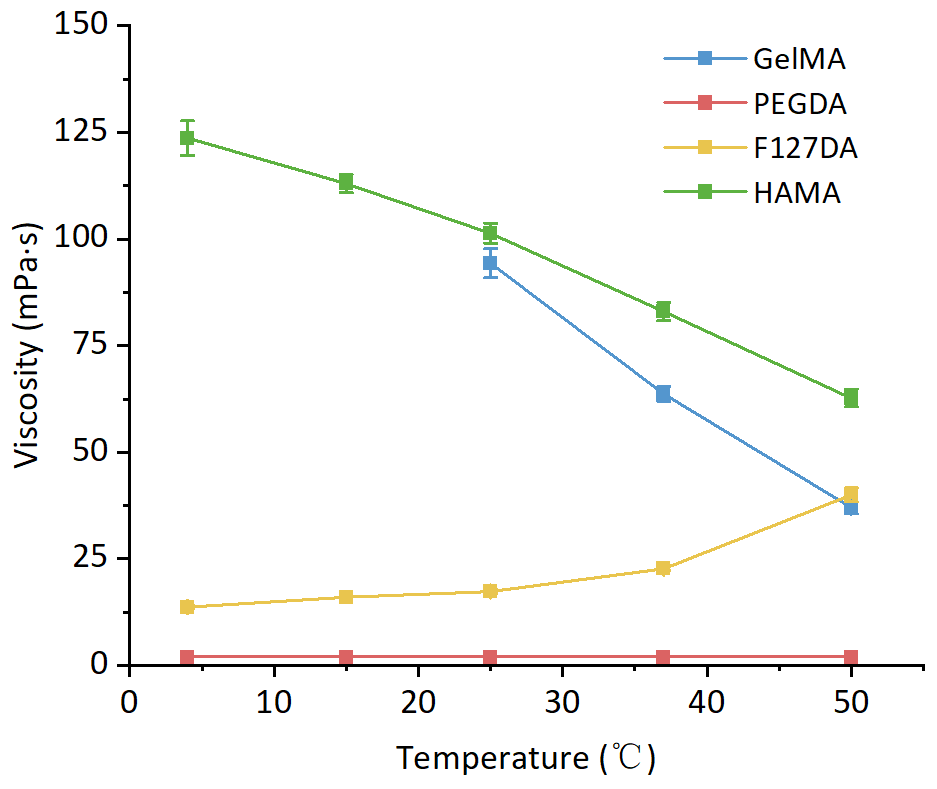


**Fig. S10**| Temperature-dependent viscosity curves of different hydrogels.


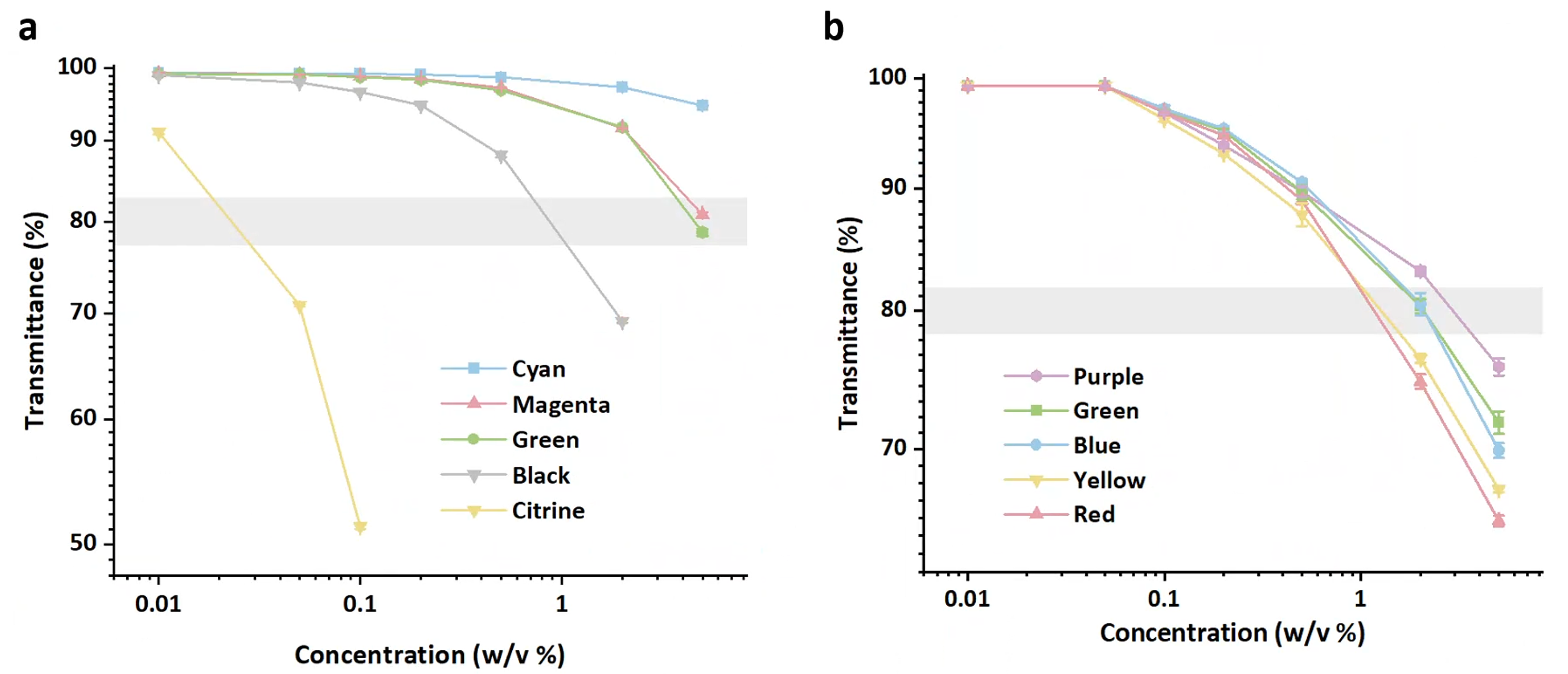


**Fig. S11|** Absorption spectra of food dyes (A) and large molecule dyes (B). The shaded area represents the optimal printing range.


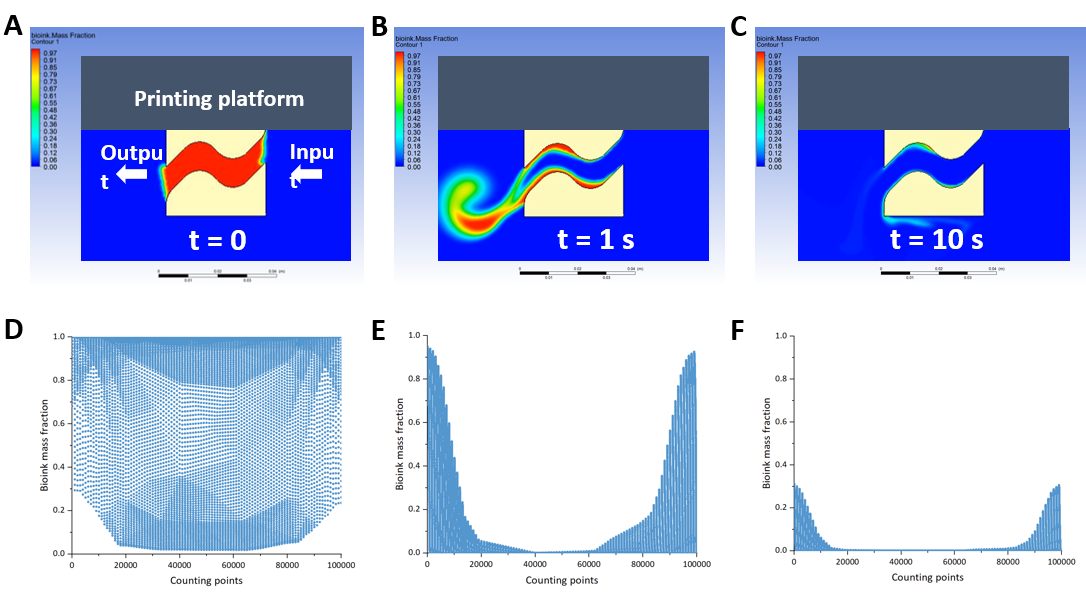


**Fig. S12**|(A) At t=0, the majority of the ink remains confined within the channel. (B) At time t = 1 s, a significant portion of the residual ink had been flushed out. (C) At time t = 10 s, the channel structure is considered to be flushed clean. Residual ink volume within the channels after rinsing for 0 s (D), 3 s (E), and 6 s (F).

**Table S1. Basic information of several hydrogels used.**

| **Materials** | Abbreviation | DoF | Molecular weight |
| --- | --- | --- | --- |
| **Gelatin methacryloy** | GelMA | 12% | ~150 kDa |
| **Hyaluronic acid methacryloyl** | HAMA | 30% | ~150 kDa |
| **Polyether F127 Diacrylate** | F127DA |  | ~15 kDa |
| **Poly (ethylene glycol) diacrylate** | PEGDA | 15% | 700 Da |
